# Review of Low-Frequency Noise Properties of High-Power White LEDs during Long-Term Aging

**DOI:** 10.3390/ma15010013

**Published:** 2021-12-21

**Authors:** Vilius Palenskis, Jonas Matukas, Justinas Glemža, Sandra Pralgauskaitė

**Affiliations:** Institute of Applied Electrodynamics and Telecommunications, Vilnius University, Saulėtekio av. 3, LT-10257 Vilnius, Lithuania; vilius.palenskis@ff.vu.lt (V.P.); justinas.glemza@ff.vu.lt (J.G.); sandra.pralgauskaite@ff.vu.lt (S.P.)

**Keywords:** electrical noise, high-power white light emitting diodes (LEDs), noise measurement technique by two-color photodetectors, optical noise, simultaneous cross-correlation coefficient

## Abstract

Low-frequency noise investigation is a highly sensitive and very informative method for characterization of white nitride-based light-emitting diodes (LEDs) as well as for the evaluation of their degradation. We present a review of quality and reliability investigations of high-power (1 W and 3 W) white light-emitting diodes during long-term aging at the maximum permissible forward current at room temperature. The research was centered on the investigation of blue InGaN and AlInGaN quantum wells (QWs) LEDs covered by a YAG:Ce^3+^ phosphor layer for white light emission. The current-voltage, light output power, and low-frequency noise characteristics were measured. A broadband silicon photodetector and two-color (blue and red) selective silicon photodetectors were used for the LED output power detection, which makes it possible to separate physical processes related to the initial blue light radiation and the phosphor luminescence. Particular attention was paid to the measurement and interpretation of the simultaneous cross-correlation coefficient between electrical and optical fluctuations. The presented method enables to determine which part of fluctuations originates in the quantum well layer of the LED. The technique using the two-color selective photodetector enables investigation of changes in the noise properties of the main blue light source and the phosphor layer during the long-term aging.

## 1. Introduction

Top-quality light sources are of high interest in industrial, science, medicine, military, and daily life applications. Light-emitting diodes (LEDs) are smaller, cheaper, operate at lower power, and have longer lifetime than other light sources (lamps or lasers) [1,2]. The simplicity and high reliability of LEDs make them an attractive choice for short-distance, moderate-speed optical data links, or for visible light communication systems [3,4,5,6]. In the most cases, investigation of the quality and reliability of LEDs is based on analyses of the current-voltage characteristic or lumen maintenance changes during the accelerated aging [1,7,8,9,10]. It has been shown that low-frequency noise characteristics also provide valuable information on semiconductor devices quality and reliability.

Four main types of noise in semiconductors and also LEDs can be distinguished: thermal (Johnson or Nyquist) noise, shot noise, generation-recombination (g-r), and 1/*f* (or flicker) noise. Thermal noise is caused by the equilibrium thermal motion, while random and independent events of charge carriers crossing a potential barrier result in shot noise. Both thermal and shot noise are characterized by a “white” spectrum, i.e., are frequency independent. Various defects and imperfections act as charge carrier capture or release centers (g-r centers or traps) and are characterized by the trapping level in the bandgap, the relaxation time and have Lorentzian-type spectrum [11]. If the relaxation times of these independent g-r processes are different and widely distributed, then superposition of Lorentzian-type spectra of a lot of such carrier capture and release processes gives 1/*f*-type noise spectrum. However, despite the fact that investigation of the nature of flicker noise has old traditions and deep roots going back many decades, origin of the 1/*f* noise is still a subject of discussion [12,13]. Moreover, when investigation of such devices as LEDs or laser diodes is carried out, not only electrical fluctuations should be analyzed, but also optical noise (light output power fluctuations) should be measured. What makes low-frequency noise investigation very attractive for LED characterization is that noise measurements are performed under normal bias conditions near the equilibrium state and are nondestructive [11].

**Figure 1:**
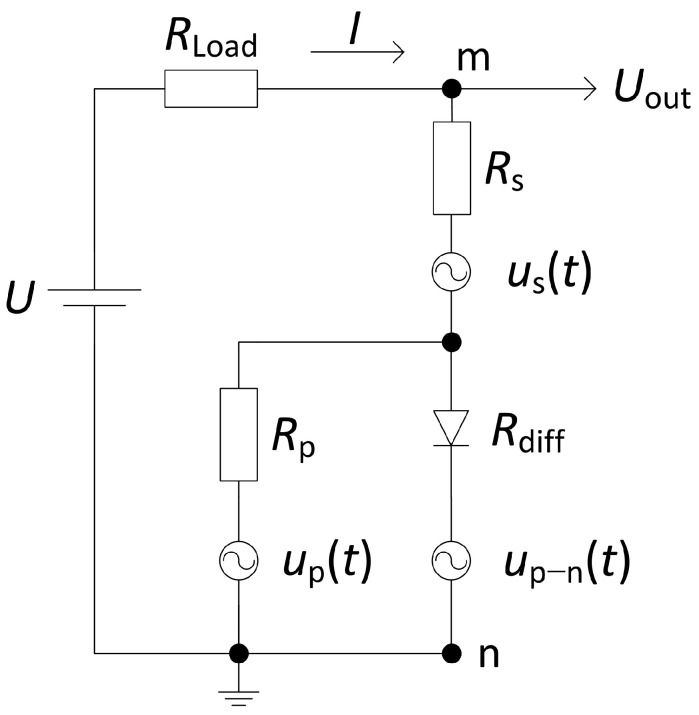
Equivalent electrical circuit of LED with voltage noise sources (*R*_Load_ >> *R*_mn_).

In order to explain the low-frequency noise properties of LED, firstly, the equivalent electrical circuit of LED with voltage noise sources, which is proposed in Figure 1, should be discussed. Here, the resistance *R*_p_, which is parallel to the differential resistance of the LED *R*_diff_ = (d*U*_p–n_/d*I*), represents the leakage currents and is not necessarily linear [14]. The *R*_s_ represents the series resistance of the material near the contacts and the contacts’ own resistance. The low-frequency noise sources located in every mentioned part of the equivalent LED circuit are described as voltage fluctuation sources: *u*_p_(*t*), *u*_p–n_(*t*) and *u*_s_(*t*). Usually, at small bias, the leakage resistance shunts the differential LED resistance, and this results in the dominance of the current leakage caused noise at small forward currents. Considering that p-n junction current increases exponentially with the applied voltage, it quickly exceeds the leakage channel current. Therefore, the noise source that represents the voltage fluctuations in p-n junction region prevails over the leakage channel noise at moderate current level. Influence of the fluctuations related to both contact regions arises only at high current (bias) values, when the voltage drop on the series resistance is comparable to the voltage drop on the p-n junction *U*_p–n_. Despite the fact that origin of the leakage current may vary in different LEDs, such equivalent electrical circuit makes it easy to describe the noise sources in various LEDs for different bias values.

Investigation of noise characteristics, especially the g-r and the 1/*f* fluctuations, of various semiconductor devices is valuable not only as noise level evaluation, but this is also a highly sensitive and informative method for clarifying physical processes that occur during device degradation and for predicting device quality and reliability [11,12,15,16,17]. Noise characteristics of nitride-based light-emitting diodes have been investigated and compared with the LEDs based on other material. Comparison of noise characteristics of LEDs based on various materials (such as GaAs, AlGaAs, GaAsInP, GaP, InAsSb, GaN, InGaN) shows a tendency that nitride-based diodes have about an order of magnitude larger electrical noise intensity compared with LEDs based on other materials [18,19,20]. The results indicate that LED structures with InGaN and GaN layers contain more defects, which modify current flow through the device, than LEDs with GaP or GaAlAs layers. High-level electrical fluctuations, which are not related to the light intensity fluctuations, show presence of defects in the contact or pre-contact regions but not in the active area of LED [21,22]. These defects cause a non-homogeneous current flow through the contacts, i.e., current flows through the separate channels formed by such defects; this leads to fluctuations of the diode resistance and increases 1/*f* noise intensity. Variations in the LED’s current-voltage and output light power characteristics during the aging correlate with changes of the noise characteristics. Analysis of the noise characteristics demonstrate that low-frequency noise measurement results can be used for device lifetime estimation. It is found that LEDs, which, under reverse bias, have quite low electrical 1/*f* type noise, have smaller reverse current, larger breakdown voltage, and longer operation lifetime compared with those that demonstrate more intensive 1/*f* fluctuations [18]. Physical processes that cause LED degradation also lead to the intensive 1/*f* type noise. The LED structures that contain macro-defects have larger leakage current, enhanced non-radiative recombination, and more rapid device degradation what cause shorter operation lifetime, especially at accelerated aging conditions [15,17,23,24,25]. The above-mentioned defects can be formed during LED fabrication or obtained during device degradation. In all cases, the presence of defects in LED structure causes shorter device lifetime, and these issues can be detected by measurement of the electrical and optical fluctuations and their correlation.

The low-frequency electrical and optical noise characteristics of white nitride-based LEDs, their quality, and their reliability during long-term aging at normal operation condition at room temperature (RT) were extensively investigated in [18,26,27,28,29,30]. There are studies of other research groups where low-frequency noise and degradation mechanisms of nitride-based LEDs during aging have been analyzed [15,17,31,32,33,34]. The conditions and types of different aging experiments performed together with the low-frequency noise investigation are presented in Table 1. There are not many scientific papers that focus on the long-term aging under normal operation conditions and, especially, on the white LEDs investigation as they contain an additional element—the phosphor layer. In this short review, we present a comprehensive summary of our investigation of electrical and optical (output light power) fluctuations, their cross-correlation coefficient of high power (1 W and 3 W) phosphor-converted white LEDs, and noise characteristics related to LED degradation during long-term aging.

## 2. Details of Investigated High-Power White LEDs and Low-Frequency Noise Measurement Technique

Summary of the research results presented in this paper are centered on the investigation of white LEDs, fabricated based on blue InGaN and AlInGaN QW diodes with the surface covered by a YAG:Ce^3+^ phosphor layer emitting broad yellow light.

**Figure 2:**
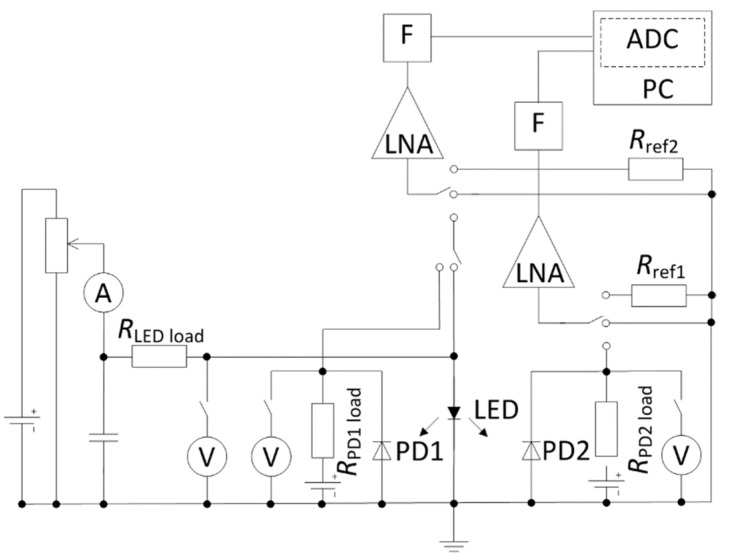
Noise measurement circuit: ADC, an analog−digital converter; F, a bandpass filter; LNA, a low-noise amplifier; PC, a personal computer; PD1 and PD2, photodetectors; *R*_LED load_, *R*_PD1 load_, *R*_PD2 load_, load resistances; *R*_ref1_, *R*_ref2_, reference resistors.

Optical spectra of the emitted light were measured by Optical Spectrum Analyzer Advantest Q8341 with 0.01-nm resolution in the wavelength range from 350 nm to 1000 nm. The noise measurement circuit is presented in Figure 2. The current-voltage characteristics, light output power, and noise characteristics were measured at room temperature in a wide forward current range. Considering the noise characterization of optoelectronic devices, it is important to measure simultaneously both optical noise (the output light power fluctuations detected by photodiode) and electrical noise (the LED terminal voltage fluctuations). We used the Cooley–Tukey Fast Fourier Transform algorithm for calculation of the noise spectra in the frequency range from 10 Hz to 20 kHz. The own noise of the measurement system has been eliminated by short-circuiting the input of low-noise amplifiers. The absolute value of the voltage noise spectral density has been calculated by comparison with the thermal noise of the reference resistance *R*_ref_:

(1) $$S_{u\;\mathrm{el}}=\frac{\langle u_{\mathrm{LED}}^{2}\left(t\right)\rangle -\langle u_{\mathrm{syst}}^{2}\left(t\right)\rangle }{\langle u_{\mathrm{ref}}^{2}\left(t\right)\rangle -\langle u_{\mathrm{syst}}^{2}\left(t\right)\rangle }4kT_{0}R_{\mathrm{ref}},$$

where $\langle u_{\mathrm{LED}}^{2}\left(t\right)\rangle$, $\langle u_{\mathrm{ref}}^{2}\left(t\right)\rangle$ and $\langle u_{\mathrm{syst}}^{2}\left(t\right)\rangle \;$are, respectively, variances of the noise signals of the LED, thermal noise of the reference resistor, and the measurement system in the narrow frequency band ∆*f*; *T*_0_ is absolute temperature of the reference resistor. Similar estimation of the spectral density of optical noise has been estimated by changing the variance $\langle u_{\mathrm{LED}}^{2}\left(t\right)\rangle$ with the variance of the photodetector measured voltage fluctuations $\langle u_{\mathrm{PD}}^{2}\left(t\right)\rangle$.

**Figure 3:**
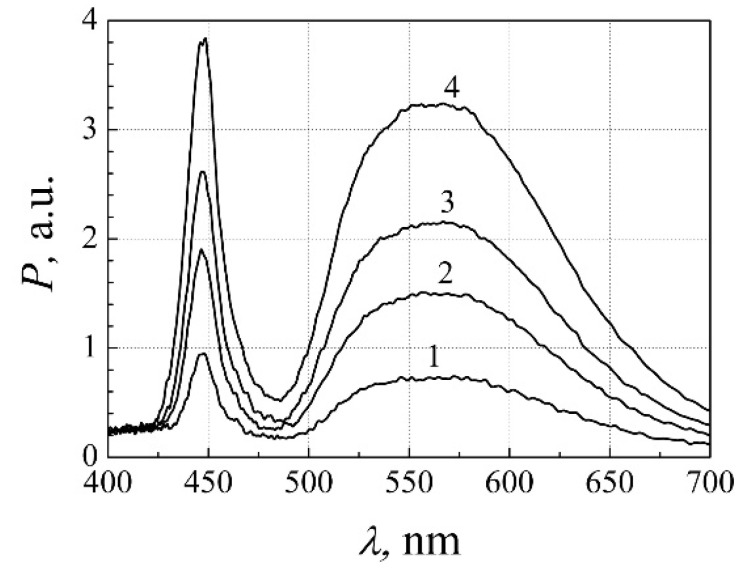
Typical optical spectra of AlInGaN white LED before aging at different forward currents *I*, mA: 1–21.5, 2–55.5, 3–80.0, and 4–136.9. Reprinted with permission from [26].

The emitted light power and its fluctuations were measured mainly by the broadband silicon photodetector with an effective surface area of 16 mm^2^. A typical optical spectrum of the investigated white LEDs has two peaks (Figure 3): the blue light emitted by QWs of a semiconductor chip and the broadband yellow light emitted by YAG:Ce^3+^ phosphor layer.

**Figure 4:**
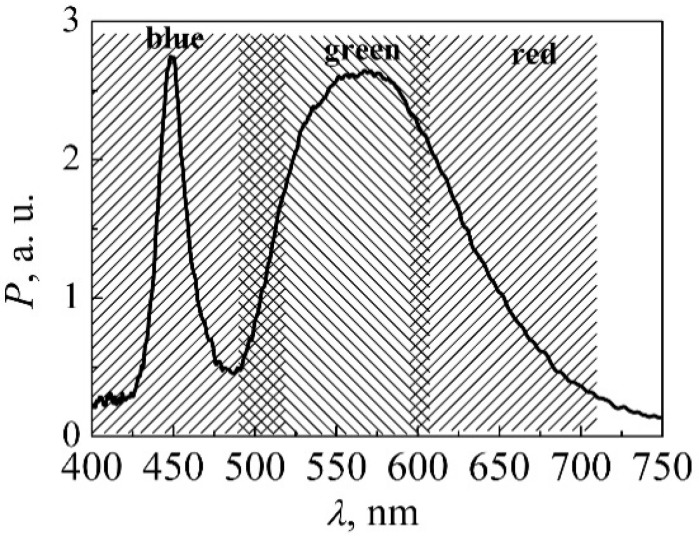
Ranges of spectral response (dashed areas) of each photodetector of the photodiode matrix S9702 and the light spectrum of white LED at 300 mA current (solid line). © 2011 IEEE. Reprinted with permission from [28].

In this paper, we also present results from research in which the LED output light power and optical noise signal were measured by the photodiode matrix (Hamamatsu S9702) that has three photodiodes (PDs), which are sensitive to different ranges of visible spectrum (Figure 4): (1) the sensitivity maximum of the first PD coincides with the blue light peak (460 nm), (2) the peak sensitivity of the second PD is at 540 nm, and (3) the third PD has a maximum sensitivity at 620 nm. The effective area of photosensitive surface is 1 mm^2^. Using such a photodiode matrix enables a separation and examination of different parts of the white LED optical spectrum: blue light (BL) photodiode is sensitive to the optical signal from the active region of QWs and red light (RL) PD is suitable for investigation of the luminescence light from the phosphor layer. Such separation of the radiation spectrum enables determination of physical processes that participate in different parts of the white LED structure and which of them has the highest influence on degradation. Optical and electrical noises and their simultaneous cross-correlation coefficient; also, cross-correlation between two optical signals, detected by the BL and RL photodetectors, were measured at room temperature.

Special attention was pointed to measurement and interpretation of the cross-correlation coefficient between electrical and optical fluctuations. In order to evaluate the correlation coefficient, measurements of the electrical and optical fluctuations were performed simultaneously; i.e., processing of both noise signals was produced with two identical channels having identical low-noise amplifiers, filter systems, and analog-digital converter (National Instruments^TM^ PCI-6115 card) (Figure 2). The simultaneous cross-correlation coefficient was directly measured not only over the frequency range from 10 Hz to 20 kHz but also in every one-octave frequency range with one-octave digital filters having the following central frequencies *f*_c_ (Hz): 15, 30, 60, 120, 240, 480, 960, 1920, 3840, 7680, and 15,360.

**Figure 5:**
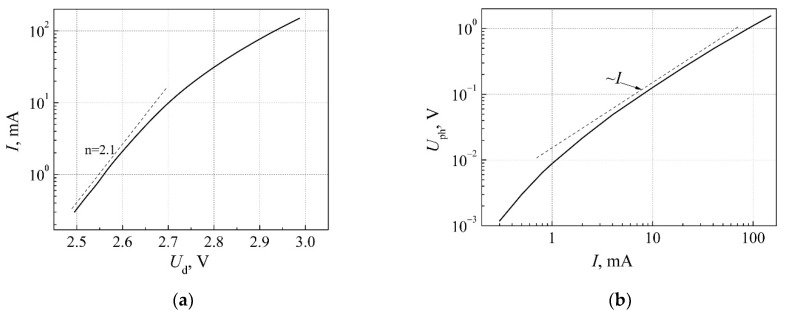
(**a**) Typical current−voltage characteristic (*n* is the non-ideality factor); (**b**) typical photodetector voltage (proportional to the emitted light power) dependence on d.c. current of white LED. Reprinted with permission from [21].

A typical current-voltage characteristic of white LED is shown in Figure 5a. Usually, the non-ideality factor *n* of the current-voltage characteristic $I=I_{0}\left(\exp\left[qU/\left(nkT\right)\right]-1\right)$ is very close to the value specific for the charge carrier recombination process in p-n junction. Deviation from the exponential dependence at higher current is due to the voltage drop in a series resistance of the LED. A typical dependence of emitted white light intensity on LED d.c. current is presented in Figure 5b: here, the silicon photodetector voltage is proportional to the total emitted light output power.

## 3. Analysis of White LED Properties by Noise Correlation Method

**Figure 6:**
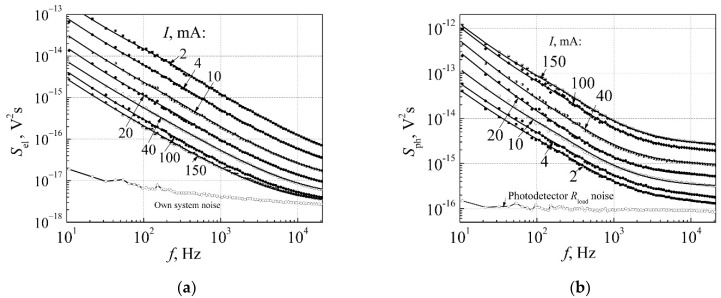
Electrical (**a**) (dots are experimental results, solid lines are calculated by Equation (2)) and optical (**b**) (dots are experimental results, solid lines are calculated by Equation (3)) noise spectra of white 3 W InGaN LED at different forward currents before aging (optical noise has been measured by the broadband silicon photodetector). Reprinted with permission from [21].

Power spectral densities of electrical and optical fluctuations of light-emitting diodes at low frequencies can be represented as a sum of independent components of 1/*f*, 1/*f*^α^, Lorentzian, and shot or thermal noise spectra:

(2) $$S_{\mathrm{el}\;\mathrm{total}}\left(f\right)=\frac{A_{\mathrm{el}\;1/f}}{f}+\frac{A_{\mathrm{el}\;1/f^{\alpha }}}{f^{\alpha }}+\frac{A_{\mathrm{el}\;\mathrm{gr}}\tau }{1+{\left(2\pi f\tau \right)}^{2}}+S_{\mathrm{el}\;\mathrm{shot}};$$

(3) $$S_{\mathrm{ph}\;\mathrm{total}}\left(f\right)=\frac{A_{\mathrm{ph}\;1/f}}{f}+\frac{A_{\mathrm{ph}\;1/f^{\alpha }}}{f^{\alpha }}+\frac{A_{\mathrm{ph}\;\mathrm{gr}}\tau }{1+{\left(2\pi f\tau \right)}^{2}}+S_{\mathrm{ph}\;\mathrm{shot}};$$

where quantities *A_j_* describe the intensities of noise components. A number of spectral components *j* depends on the complexity of spectrum. Such noise spectrum presentation is very useful for further analysis of noise properties because it means that noise sources with 1/*f*, 1/*f*^α^, and Lorentzian-type spectra are statistically independent. The experimental result (dots) approximated by Equations (2) and (3) are shown in Figure 6a,b by solid lines. 

The simultaneous cross-correlation coefficient between electrical and optical fluctuations is estimated by such expression:

(4) $$r=\langle u_{\mathrm{el}\;\mathrm{total}}\left(t\right)\cdot u_{\mathrm{ph}\;\mathrm{total}}\left(t\right)\rangle /{\left(\sigma_{\mathrm{el}\;\mathrm{total}}^{2}\cdot \sigma_{\mathrm{ph}\;\mathrm{total}}^{2}\right)}^{1/2},$$

where brackets <…> mean averaging both on time and on number of realizations, and $\sigma_{\mathrm{el}\;\mathrm{total}}^{2}=<u_{\mathrm{el}\;\mathrm{total}}^{2}\left(t\right)>$, $\sigma_{\mathrm{ph}\;\mathrm{total}}^{2}=<u_{\mathrm{ph}\;\mathrm{total}}^{2}\left(t\right)>$ are, respectively, the total variances of electrical and optical fluctuations.

For further interpretation of the results, the correlation function can be presented in the following way:

(5) $$\langle u_{\mathrm{el}\;\mathrm{total}}\left(t\right)\cdot u_{\mathrm{ph}\;\mathrm{total}}\left(t\right)\rangle =\sum_{j=1}^{3}\langle u_{\mathrm{el}\;j}\left(t\right)\cdot u_{\mathrm{ph}\;j}\left(t\right)\rangle ,$$

where index *j* defines three correlation function components for 1/*f* (*j* = 1), 1/*f*^α^ (*j* = 2), and Lorentzian (*j* = 3) type fluctuations, considering that the shot, the thermal, and the measurement system’s own noise components are uncorrelated.

It is well known that the correlation function reflects the linear relation between two random processes; thus, each component of the low-frequency optical noise can be written as

(6) $$u_{\mathrm{ph}\;j}\left(t\right)=a_{j}u_{\mathrm{el}\;j}\left(t\right),$$

where *a_j_* is the coefficient of proportionality. Besides, the quantity *a_j_* has meaning of the modulation coefficient of the emitted light power modulation by the LED current fluctuations. Thus, the simultaneous correlation function $k_{j}\left(t,t\right)$ between optical and electrical noise components can be described as

(7) $$k_{j}\left(t,t\right)=\langle u_{\mathrm{ph}\;j}\left(t\right)\cdot u_{\mathrm{el}\;j}\left(t\right)\rangle =a_{j}\langle u_{\mathrm{el}\;j}\left(t\right)\cdot u_{\mathrm{el}\;j}\left(t\right)\rangle =a_{j}\sigma_{\mathrm{el}\;j}^{2};$$

(8) $$\sigma_{\mathrm{ph}\;j}^{2}=a_{j}^{2}\sigma_{\mathrm{el}\;j}^{2}.$$

From Equations (7) and (8), we have the following:

(9) $$a_{j}={\left(\sigma_{\mathrm{ph}\;j}^{2}/\sigma_{\mathrm{el}\;j}^{2}\right)}^{1/2}$$

and simultaneous cross-correlation function can be described as

(10) $$k_{j}\left(t,t\right)=\pm {\left(\sigma_{\mathrm{ph}\;j}^{2}\cdot \sigma_{\mathrm{el}\;j}^{2}\right)}^{1/2}.$$

The sign of the correlation function is determined by the sign of *a_j_*. In a common case, not all low-frequency electrical fluctuation components (for example, with 1/*f*, 1/*f*^α^, or with Lorentzian-type spectra) are completely correlated with the optical fluctuations: contact or electrical noise sources in the passive layers of LED do not provoke intensity fluctuations in the emitted light. Therefore, each spectral density component of the low-frequency electrical noise can be written as a sum of correlated and uncorrelated parts:

(11) $$S_{\mathrm{el}\;j}\left(f\right)=S_{\mathrm{el}\;j\;cor}\left(f\right)+S_{\mathrm{el}\;j\;uncor}\left(f\right)=d_{j}S_{\mathrm{el}\;j}\left(f\right)+\left(1-d_{j}\right)S_{\mathrm{el}\;j}\left(f\right);$$

where parameter *d_j_* shows which part of the spectral component $S_{\mathrm{el}\;j}\left(f\right)$ of electrical noise causes the emitted light power fluctuations. Thus, the simultaneous cross-correlation coefficient (4), according to Equations (7)–(11), can be expressed as

(12) $$r=[\sum_{j=1}^{3}\left(d_{j}\sigma_{j\;\mathrm{el}}^{2}\cdot \sigma_{j\;opt}^{2}{)}^{1/2}\right]/{\left(\sigma_{\mathrm{el}\;\mathrm{total}}^{2}\cdot \sigma_{\mathrm{ph}\;\mathrm{total}}^{2}\right)}^{1/2}.$$

Similar expression can be written for simultaneous cross-correlation coefficient for each one-octave frequency band.

**Figure 7:**
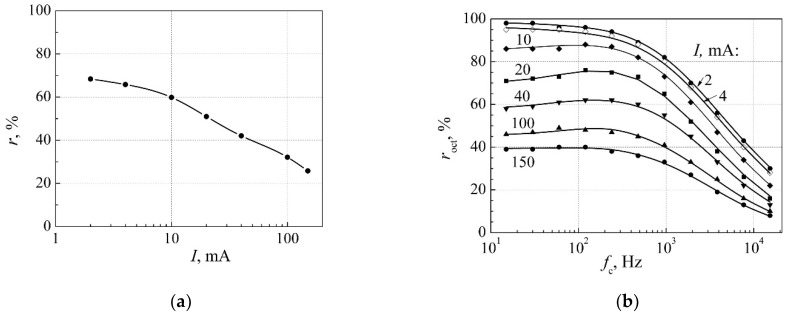
(**a**) Dependence of the cross-correlation coefficient *r* between electrical and optical fluctuations on current of 3 W white LED in the whole investigated frequency range 10 Hz–20 kHz (dots are the measurement results, solid line is calculated by Equation (12)); (**b**) dependence of the cross-correlation coefficient *r*_oct_ between electrical and optical fluctuations in the one-octave frequency band on the central frequency *f*_c_ of the octave filter at different forward currents (dots are the measurement results, solid lines are calculated by Equation (12) for each one-octave filter band). Reprinted with permission from [21].

An example of dependence of the cross-correlation coefficient *r* between electrical and optical fluctuations on forward current for 3 W white InGaN LED is presented in Figure 7a. The quantities *d_j_* were defined by the comparison of the experimental data of the cross-correlation coefficient with the calculated one by Equation (12). It is seen that there is a very good agreement between experimental and calculation results. Dependence of the cross-correlation coefficient *r*_oct_ on the central frequency of octave filter *f*_c_ at different currents is presented in Figure 7b. Decrease of the correlation coefficient *r*_oct_ at higher frequencies is due to the larger contribution of noise with constant spectral density. The obtained results show that the correlation coefficient decreases with d.c. current increasing. Here, in every octave, the quantities *d_j_* are the same; they only depend on the type of the low-frequency fluctuations and on d.c. current of the LED.

**Figure 8:**
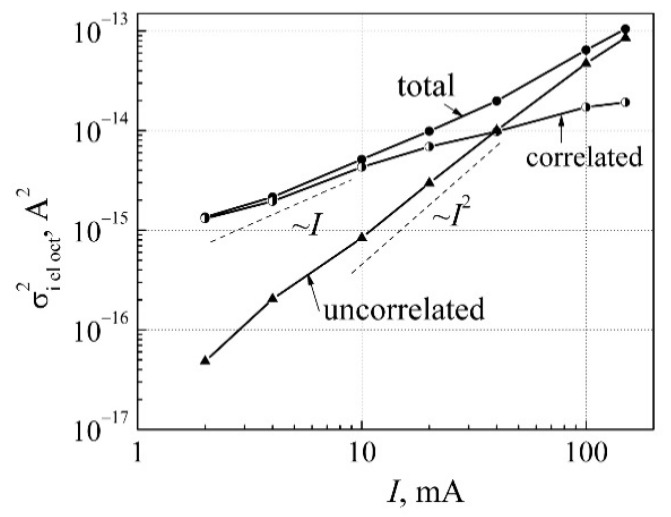
Dependence of the current fluctuation variance (total, correlated, and uncorrelated parts of the low-frequency electrical noise) on forward current in the one−octave frequency band with the central frequency of 240 Hz.

To clear up the origins of the low-frequency noise components and their locations in the LED structure, and considering that light emission is caused by the charge carrier radiative recombination in the quantum wells, it is convenient to present the electrical voltage fluctuations variance ($\sigma_{u\;\mathrm{el}}^{2}$) as the current variance ($\sigma_{i\;\mathrm{el}}^{2}$):

(13) $$\sigma_{i\;\mathrm{el}}^{2}=\sigma_{u\;\mathrm{el}}^{2}/R_{\mathrm{diff}}^{2},$$

where *R*_diff_ is the differential resistance of the LED. Variance components (total, correlated, and uncorrelated) of the current fluctuations of the white LED in the one-octave filter with the central frequency of *f*_c_ = 240 Hz are presented in Figure 8. Variance of the total current fluctuations is approximately proportional to the forward current. This is characteristic for the low-frequency noise in semiconductor devices (noise source *u*_p–n_(*t*) in Figure 1) [21]. Considering that the light emission can occur only in the active area of the LED, it can be stated that correlated electrical and optical fluctuations are due to the random charge carriers capture processes in the localized states in the QWs region. Capture of the charge carrier events create random potential fluctuations of the quantum wells, which modulate that part of charge carriers that recombine in QWs and produce photons. Variance of the uncorrelated part of the current fluctuations is ~ *I* ^2^, which is characteristic for the peripheral region of LEDs, i.e., defects, which are located outside the QWs, e.g., in the *n*-GaN or *p*-GaN layers, contacts, or surface (noise sources *u*_s_(*t*) and *u*_p_(*t*) in Figure 1). The uncorrelated part of the current fluctuations has no influence on the light power fluctuations.

The presented technique enables determination of the cross-correlation coefficient dependences on both forward current and frequency and estimation of which part of the electrical fluctuations produces optical fluctuations in the active area of the LED. Figure 6 and Figure 7 show that the presented cross-correlation analysis method is valid and suitable for the LED analysis as long as the approximation of the experimental spectra by Equations (2) and (3) is done carefully and accurately. This method was also applied for analysis of noise characteristics of red, green, and blue light LEDs [20,22,23,35,36]. Based on the investigations, it can be summarized that at small currents (<10 mA), the low-frequency optical and electrical noise components are strongly correlated; the current mostly flows through the active LED region, and the number of the emitted photons is modulated by the random charge carrier capture processes in localized defect states in the active QWs layers. At higher forward currents, the uncorrelated optical and electrical noise components prevail, which has a great impact on the level of total electrical fluctuations; however, it does not significantly influence the light output power fluctuations.

## 4. Influence of Light Power Incident on Photodetector on the Low-Frequency Optical Noise Level

**Figure 9:**
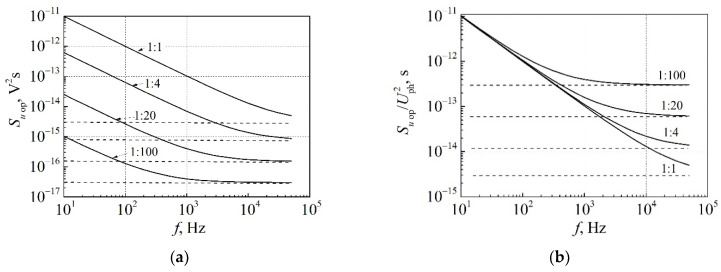
(**a**) The optical noise spectra and (**b**) the relative optical noise spectra at various incident light attenuation ratios at constant LED bias regime (*I*_LED_ = 140 mA).

In the course of the investigation of the optical noise and the cross-correlation between optical and electrical fluctuations, the question arises: how does the amount of the light incident on the photodetector influence the noise measurement results? Similar issue can be encountered when photodetectors with different effective surface area are used. The amount of the light incident on the surface of the photodetector at a constant bias of the LED can be changed using light attenuation filters. The optical noise spectra at various incident light attenuation ratios are presented in Figure 9a. Level of the optical shot noise (dashed lines) decreases linearly with incident light intensity decreasing as the shot noise is proportional to the rate of incident photons. The spectral density of the measured low-frequency optical noise (1/*f* noise, solid lines) decreases more steeply with the incident light attenuation. The relative optical noise spectral densities $S_{u\;\mathrm{op}}/U_{\mathrm{ph}}^{2}$ are presented in Figure 9b, considering the linear dependence of the photodetector photovoltage $U_{\mathrm{ph}}^{}$ on the incident light power. It is clearly seen that the relative optical noise spectral density at low frequencies does not depend on the intensity of the incident light. This proves once again that the low-frequency noise is of modulation type; random captures of charge carriers in the active region of the LED modulate the emitted light power. The same tendency can be observed with a decrease of the effective surface area of the photodetector: the smaller the effective surface of the photodetector, the more clearly the shot noise will be expressed and more effectively the low-frequency noise components (1/*f*, 1/*f*^α^ and generation-recombination) will be attenuated.

Attention should be also paid to the validation of the cross-correlation coefficient measurement results. If *γ* is the ratio between the light power incident on the photodetector surface and the total emitted light power, then the relative cross-correlation coefficient can be expressed as

(14) $$r=\langle u_{\mathrm{el}\;\mathrm{total}}\left(t\right)\cdot \gamma \;u_{\mathrm{ph}\;\mathrm{total}}\left(t\right)\rangle /{\left(\sigma_{\mathrm{el}\;\mathrm{total}}^{2}\cdot \gamma^{2}\sigma_{\mathrm{ph}\;\mathrm{total}}^{2}\right)}^{1/2}=\;=\langle u_{\mathrm{el}\;\mathrm{total}}\left(t\right)\cdot \;u_{\mathrm{ph}\;\mathrm{total}}\left(t\right)\rangle /{\left(\sigma_{\mathrm{el}\;\mathrm{total}}^{2}\cdot \sigma_{\mathrm{ph}\;\mathrm{total}}^{2}\right)}^{1/2}.$$

Though the correlation function $k\left(t,\;t\right)=\langle u_{\mathrm{el}\;\mathrm{total}}\left(t\right)\cdot \gamma \;u_{\mathrm{ph}\;\mathrm{total}}\left(t\right)\rangle$ depends on the intensity of the incident light power fluctuations, the relative cross-correlation coefficient does not depend on the light power incident on the photosensitive surface of the detector. Thus, the measurement of the relative cross-correlation coefficient by photodetectors with different effective surface areas gives the same result.

Considering that the active photodetector surface area of the photodiode matrix is about 16 times smaller than the surface area of the broadband photodetector (16 mm^2^), the initial measured optical noise level in the frequency octave of (10–20) Hz (presented in Figure 9) is about 500 times smaller than can be obtained with the white light broadband photodetector at the same current. Similarly, the shot noise is about 20 times smaller with respect to the optical spectrum range width.

## 5. Low-Frequency Noise Properties of High Power White AlInGaN LEDs during Aging

**Figure 10:**
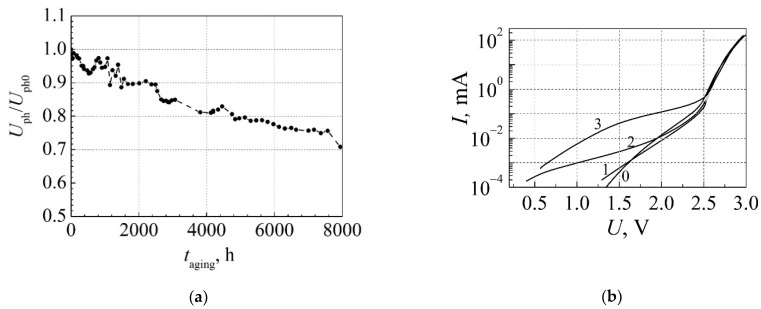
(**a**) Dependence of photovoltage (proportional to the LED output light power) on aging time measured at 30 mA current; (**b**) current–voltage characteristics after different aging time in h: 0–before aging; 1–2200; 2–2730; 3–8000.

In this section, the low-frequency noise properties of high-power AlInGaN-based white LEDs are discussed. Dependences of the output light power, the optical and electrical noises in the frequency range from 10 Hz to 20 kHz, and their simultaneous cross-correlation coefficient on forward current have been measured for initial LEDs and at particular times during aging at the maximum permissible current: *I*_max_ = 1 A. The output light power and its fluctuations were measured by the broadband silicon photodetector. The optical output power gradually decreases during all aging experiments, and after 8000 h of aging it is decreased about 30 % (Figure 10a).

**Figure 11:**
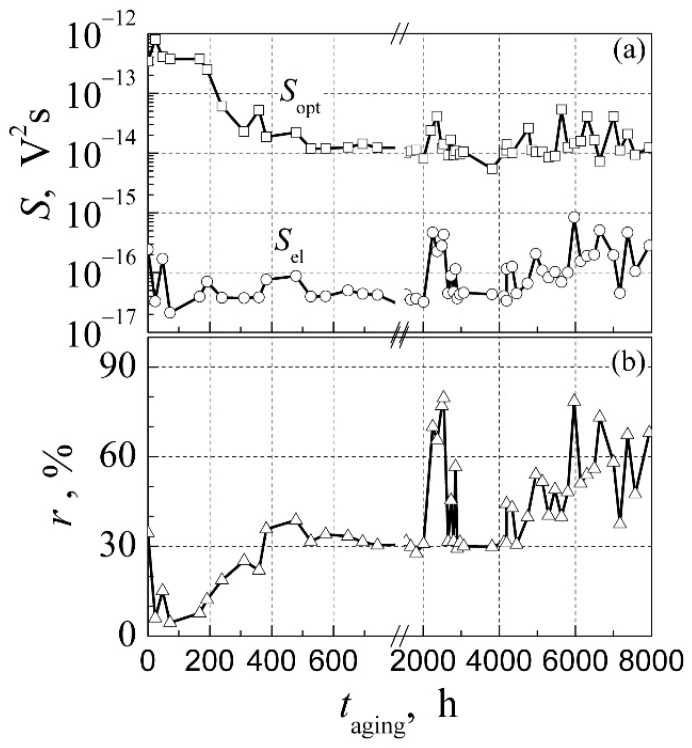
Dependences of (**a**) the electrical and optical noise spectral densities (at 280 Hz frequency) and (**b**) the simultaneous cross-correlation coefficient between optical and electrical fluctuations (in frequency range 10 Hz–20 kHz) on the aging time measured at forward 30 mA current. Reprinted with permission from [26].

The current-voltage characteristics after different aging time intervals are presented in Figure 10b. It is seen that the leakage current at low bias sharply increases (the non-ideality factor in this bias region changes from 4 at 0 h to 12 after 8000 h), which shows formation of the current leakage micro-channels in the LED structure. There is strong correlation between the leakage current at low bias and noise characteristics in the corresponding LED operation range. In the initial phase of aging (up to 400 h), the electrical noise intensity slightly decreases, while the optical noise level decreases about two orders of magnitude (Figure 11a). It shows that nonstable defects and impurities in the active layer of the LED migrate to a more stable position: some ordering of the structure occurs. The simultaneous cross-correlation coefficient during the first 100 h decreases and then increases up to 30% (Figure 11b). A steep increase of the low-frequency electrical and optical noise intensity has been observed during the long-term aging in the time interval of (2010–2500) h. These fluctuations are highly correlated: the correlation coefficient reaches 80 %. Such noise peaks indicate the generation-recombination process in the region of the quantum wells, as it has been observed in InGaAsP diode lasers with QWs [37]. During aging in the time interval from 4000 h to 8000 h, the noise intensity demonstrates unstable behavior caused by formation of localized states of defects in the barrier layers of the QWs due to migration of defects at high aging current and related to increase of the sample temperature due to Joule heating. Apparent defect migration and penetration into the LED active area due to local overheating and non-uniform charge carrier transport has been observed in blue InGaN LEDs during accelerated aging in a much shorter time interval of the experiment from 280 h to 1000 h [33].

**Figure 12:**
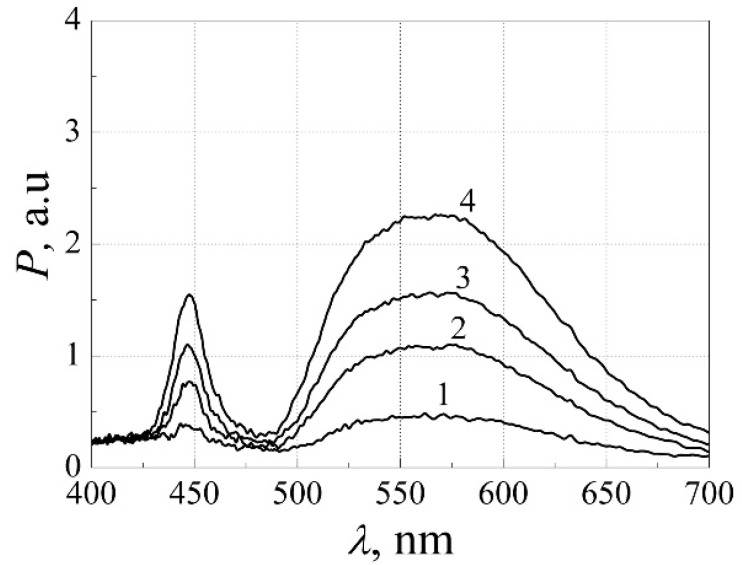
Optical spectra of the high-power white AlInGaN LED at forward currents *I*, mA: 1–20.9; 2–55.5; 3–86.4; 4–141.3 after 8000 h of aging. Reprinted with permission from [26].

The emitted light spectra after 8000 h of aging are presented in Figure 12. From the comparison of Figure 3 and Figure 12, it is observed that after the long-term aging peak of the blue light decreases more than twice, while the peak of the yellow light decreases only about 30%. Considering that the spectral range of the yellow emission is many times larger than the blue spectrum part, the total optical power characteristic during aging (Figure 10a) is governed mainly by the changes in phosphor layer luminescence intensity. The significant decrease of the blue light peak could be attributed to the increased blue light absorption in the phosphor layer.

An analysis excluding the optical shot and the measurement system noises, which are not correlated with the electrical fluctuations, shows that the low-frequency electrical and optical noise components at currents smaller than 50 mA are completely correlated. This indicates that noise sources are in the quantum well region of the investigated LEDs. During aging, only the multi-quantum well region degrades, but the phosphor layer does not. Additional noise sources are observed at larger currents, and they differently influence the electrical and optical fluctuations.

Studies on physical mechanisms of the low-frequency fluctuations in materials and devices reveal that the charge carrier capture and emission process in localized states of the defects is the main source of the low-frequency noise [12,13].

The obtained results of aging of the high-power white AlInGaN LEDs at maximum permissible current show that at the initial phase of aging (during (400–600) h) the LED structure ordering occurs, and then, during the next time interval of about 1500 h, the operation of the LED is stable; and after more (2000–4000 h) aging, a more rapid degradation of LEDs starts. After 8000 h of aging, the total light output power decreases about 30 %, while the primary blue light intensity decreases more than twice.

## 6. Investigation of the Low-Frequency Optical Noise Properties of High-Power White InGaN LEDs by Two-Color Selective Photodetectors

**Figure 13:**
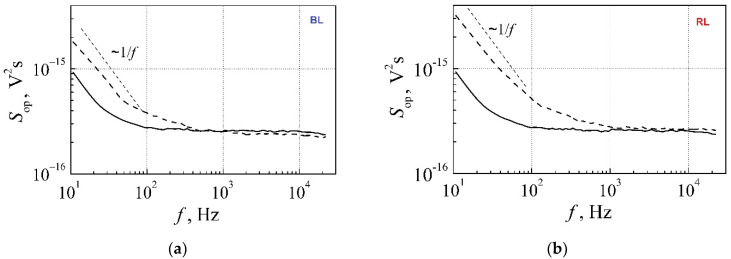
Optical noise spectra measured by (**a**) BL and (**b**) RL photodetectors at 140 mA forward current before aging (solid lines) and after 1340 h aging (dash lines) at 350 mA.

Here, the main white LEDs’ noise characteristics measured by the photodiode matrix and observed tendencies of changes of these characteristics during the long-term aging are discussed. Typical optical noise spectra measured by the blue light (BL) and the red light (RL) photodetectors are depicted in Figure 13. Properties of 1 W white LEDs have been investigated prior to the aging experiment and after 1340 h aging at the maximum permissible current (350 mA), and the summary of the optical noise measurement results for InGaN LED by BL and RL photodetectors is presented in Table 2.

Table 2 and Figure 13 demonstrate that level of the optical fluctuations at low-frequency increases after 1340 h of aging due to the growth of 1/*f* noise components in both optical noise spectra measured by BL and RL photodetectors. This also leads to the significant increase in the cross-correlation coefficient between the optical fluctuations measured by the BL and RL photodetectors (*r*_ph red-blue_) in (10–20)-Hz frequency band after the aging experiment.

**Figure 14:**
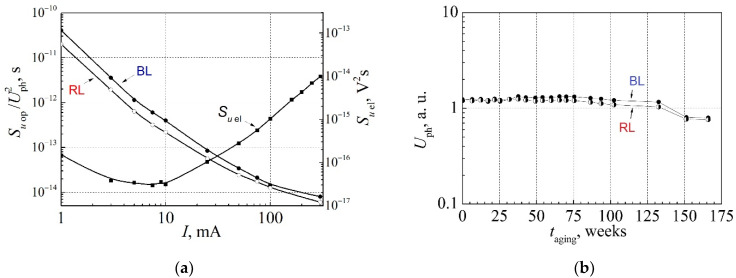
(**a**) Dependences of the electrical (right scale) and relative optical (left scale) noise spectral density on forward current at low 22 Hz frequency before aging of the high-power white LED measured by the BL photodetector (solid dots) and by the RL photodetector (open dots); (**b**) change of the light intensity measured by the BL photodetector (solid dots) and by the RL photodetector (half-filled dots) at forward 100 mA current during the long-term aging.

A deeper look should be taken at the physical processes occurring in the white LEDs during the long-term aging. For this purpose, investigation results for the high-power “cool” white multi-quantum well InGaN LEDs during the long-term aging (of total 167 weeks long) at the maximum permissible forward current (350 mA) at RT are presented. These LEDs show good linearity of the light output power dependence on the forward current. Dependences of the electrical and optical noise characteristics on the forward current before aging measured by the BL and RL photodetectors are presented in Figure 14a. The relative optical noise spectral density decreases with the forward current increase because level of the optical fluctuations is approximately proportional to the forward current. The increase of the electrical noise level at currents larger than 10 mA is related to the localized states of defects in the peripheral regions of the LED, and this electrical noise is not correlated with the emitted light fluctuations.

The light intensity (photovoltage $U_{\mathrm{ph}}^{}$ is proportional to the emitted light power measured in both blue and red light spectra regions) changes during the long-term aging are presented in Figure 14b. During 75 weeks of aging, the light intensity of the LED is almost constant, and then, it slowly decreases. After the 130-week-long aging, the emitted light power decreases more intensively, and finally, after 150 weeks, degradation of the LED reaches the threshold limit: the initial lumen output decreases about 30%.

**Figure 15:**
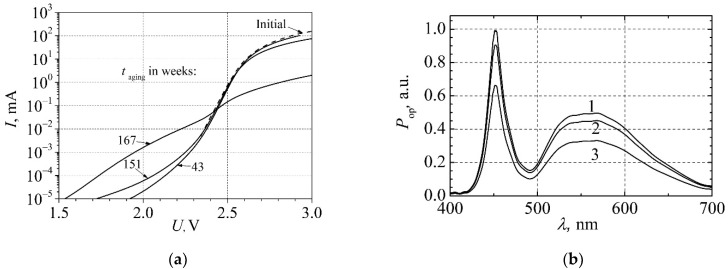
(**a**) Current−voltage characteristic of the white LED at different aging times; (**b**) optical spectra after various aging times: 1, before aging; 2, after 135 weeks, 3, after 167 weeks.

Analysis of the white LED current-voltage characteristic shows that the leakage current during the aging changes noticeably (Figure 15a). At first, it slowly increases within 150 weeks of aging; however, after 167 weeks of aging, the whole current-voltage characteristic is drastically changed. Optical spectra at different aging times are presented in Figure 15b. From the comparison of Figure 14b and Figure 15b, it is observed that the optical spectrum magnitude decreases just about 10 % during 135 weeks of aging: both the blue and the red light intensity change in the same way. It shows that the phosphor layer does not produce any additional influence on the light spectrum during the aging process: the broadband yellow light is caused by the blue light and its transformation by the phosphor layer. We have not observed any changes in the efficiency of the blue light transformation into the yellow one during aging, as it was reported in high-power AlInGaN white LEDs, and ratio between intensity of the yellow and the blue light components was approximately the same during all the experiment.

**Figure 16:**
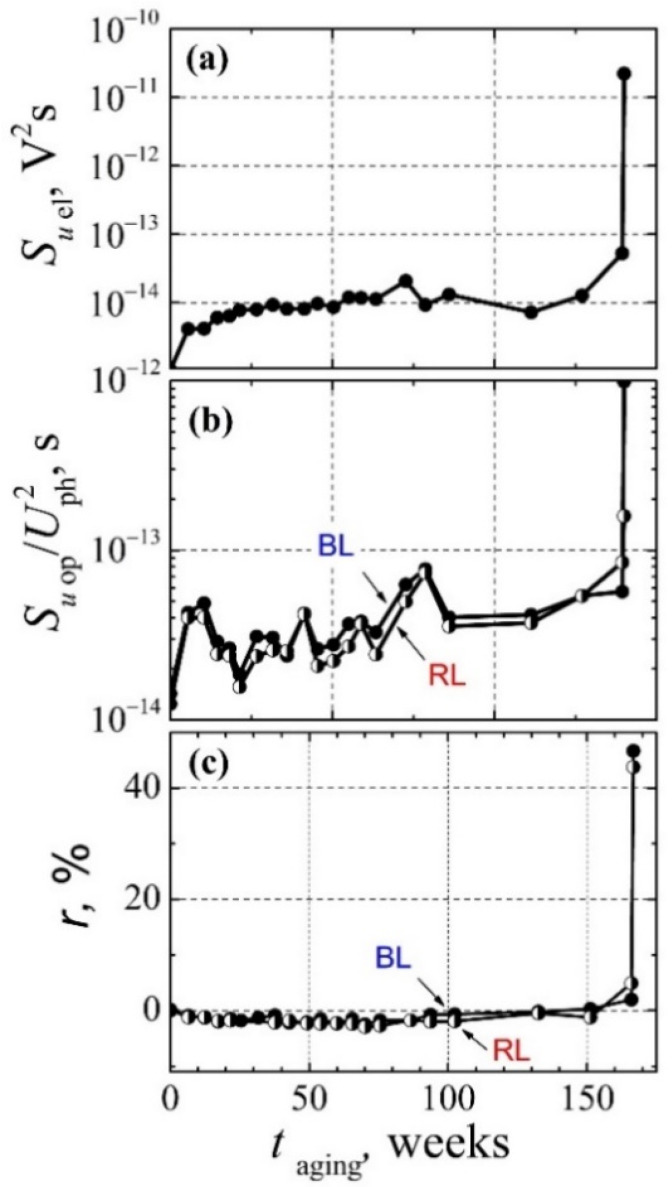
Dependences of the electrical (**a**) and the relative optical noise (**b**) spectral densities on the aging time at forward 100 mA current at 22 Hz frequency; (**c**) dependences of the cross−correlation coefficient between the electrical fluctuations and both optical blue (BL) and red (RL) light fluctuations on the aging time in the whole measured frequency range of 10 Hz–20 kHz at 100 mA current (solid dots, blue light; half-filled dots, red light).

Variation of electrical and optical fluctuations during the aging is presented in Figure 16. The electrical noise intensity slowly increases during the 166 weeks aging, and then, it drastically grows by three orders of magnitude. A similar behavior is observed for the change of the optical noise intensity (Figure 16b). The increased intensity of the electrical and the optical fluctuations; also, the greatly increased cross-correlation coefficient after the 166 weeks aging (Figure 16c) indicate that the main degradation occurs in the active area of the LED. Positive sign of the cross-correlation coefficient shows a high defectiveness of the diode QWs structure. This is also confirmed by the current-voltage characteristic and the optical spectrum behavior during aging.

There is a quite good agreement between aging results of the optical noise and the cross-correlation measured by the BL and the RL photodetectors (Figure 16b,c): the phosphor layer only reemits (transforms) a part of the blue light and, as a consequence, its fluctuations. Therefore, it can be stated that the phosphor layer does not create additional noise sources in the investigated high-power white LEDs.

When the optical shot noise and the measurement system’s own noise are eliminated from the estimation of the simultaneous cross-correlation coefficient between the optical fluctuations in the blue and the red light spectrum ranges (*r*_ph red-blue_), this cross-correlation coefficient is very close to 100%. This also explains why the coefficient *r*_ph red-blue_ after aging in Table 2 is equal to only 82% despite the high levels of the BL and the RL optical 1/*f* noise components. This confirms that low-frequency noise is originated in the quantum well layers and that phosphor luminescence does not give any additional contribution to the low-frequency noise level.

A summary of changes of the electrical and optical characteristics during the long-term aging of the white high-power AlInGaN and InGaN LEDs is presented in Table 3. Here, values of the electrical and the optical noise intensity are compared at moderate current before the series resistance-limited current is reached. Observed changes in the noise intensity during the intermediate aging stage (e.g., in the case of AlInGaN LED in (2010–2500)-h interval) demonstrate sensitivity of the low-frequency noise measurement as the LED characterization method: no major alterations during aging were observed in other electrical or optical characteristics (current-voltage, optical light power), or these changes in these characteristics became noticeable after additional aging time.

The main source of the low-frequency noise in the investigated white InGaN or AlInGaN LED is caused by the charge carrier capture and emission processes in localized states of the defects in the active layer of the LED. Changes of these states during the aging lead to the variation of the leakage current and the intensity of electrical and optical fluctuations and also the cross-correlation between optical and electrical fluctuations.

## 7. Conclusions

In this short review, we present a summary of the comprehensive investigation of characteristics of the high-power white LED with quantum wells: current-voltage characteristic, light output power and optical spectrum, low-frequency electrical and optical noise properties, and features of the cross-correlation coefficient between optical and electrical fluctuations. Additionally, LED characteristic changes during the long-term aging at the maximum permissible forward current were studied. The method based on the simultaneous cross-correlation coefficient measurement to estimate which part of the electrical noise is originated in the quantum wells layer is described. Influence of the area of the photodetector active surface on the optical noise measurement results is discussed as well: the low-frequency noise is suppressed more strongly than the shot noise when photodetector with the smaller active surface area is used. However, the relative optical noise spectral density does not depend on the intensity of the light incident on the photodetector. It is also shown that the simultaneous cross-correlation coefficient between electrical and optical fluctuations does not depend on the effective surface area of the photodetector used for optical signal detection.

The use of two photodetectors sensitive for the different light spectrum range (blue and red) enabled us to estimate the changes of the white LEDs’ initial blue light source and luminescence light of the phosphor layer during the long-term aging. The main source of the low-frequency noise in the investigated LEDs is caused by the charge carrier capture and emission processes in localized states of the defects in the quantum well layer. The phosphor layer does not cause any additional noise sources during aging.

The high sensitivity of the low-frequency noise study to nitride-based LED characterization enables future research to propose a quantitative model linking the degradation mechanism to changes in electrical and optical characteristics even in the early stage of aging.

## Figures and Tables

**Table 1:** Comparison of the different aging experiment types and conditions during which the low-frequency noise measurements of GaN-based LEDs were carried out. All samples contain QWs in their structure.

Investigated Samples	Ref.	Aging Experiment	
		Type	Conditions
InGaN/GaN	[34]	accelerated—higher current;	at RT, total of 100 h;
Blue InGaN/GaN	[15]	accelerated—higher temperature;	at 240 °C temperature, total of 300 h;
	[15]	normal operation;	at RT, total of 2500 h;
	[31,33]	accelerated—higher current ant temperature;	at 100 °C temperature, (10–2000) h;
Green InGaN/GaN	[17]	normal operation;	at RT, total of 1000 h;
Near-UV InGaN/GaN	[32]	accelerated—higher current ant temperature;	at 110 °C temperature, total of 1000 h;
White (phosphor-converted)	[26,27,28,29,30]	normal operation;	at RT, up to 167 weeks.

**Table 2:** Summary of the 1 W white InGaN LED the optical noise measurements by BL and RL photodetectors at 140 mA forward current.

Aging State	Parameters	BL Photodetector	RL Photodetector
Before aging	$U_{\mathrm{ph}}$	93 mV	125 mV
	$S_{\mathrm{op}}$ at 10 Hz	0.91·10^−15^ V^2^s	0.91·10^−15^ V^2^s
	$S_{\mathrm{op}}/U_{\mathrm{ph}}^{2}$ at 10 Hz	1.06·10^−13^ s	0.58·10^−13^ s
	$r_{\mathrm{ph}\;\mathrm{red}-\mathrm{blue}}$ (10–20) Hz	61%	
After 1340 h of aging at 350 mA	$U_{\mathrm{ph}}$	87 mV	123 mV
	$S_{\mathrm{op}}$ at 10 Hz	1.8·10^−15^ V^2^s	3.1·10^−15^ V^2^s
	$S_{\mathrm{op}}/U_{\mathrm{ph}}^{2}$ at 10 Hz	2.38·10^−13^ s	2.05·10^−13^ s
	$r_{\mathrm{ph}\;\mathrm{red}-\mathrm{blue}}$ (10–20) Hz	82%	

**Table 3:** Comparison of changes of the electrical and optical noise characteristics of the high-power white AlInGaN and InGaN LEDs during the long-term aging at RT.

Characteristic	AlInGaN LED			InGaN LED			Remark
	Aging State						
	Initial	Intermediate	Final	Initial	Intermediate	Final	
Current-voltage	Slight increase in leakage current	Leakage current increases	Strong increase in leakage current	Very slight increase in leakage current	Leakage current and *R*_s_ increases	Drastically changes in the whole range	Large *R*_s_ increase was observed in [15] after 300 h of accelerated thermal stress due to effective doping decrease of the *p*-doped layer
Optical power	Decreases about 9%	Gradually decreases	Decreases about 30%	Stable, after about half of aging time starts to decrease		Decreases about 30%	
Electrical noise	Slightly decreases up to one order of magnitude	No major changes, steep increase in (2010–2500)-h period	Unstable behavior followed by electrical noise increase	Increases up to one order of magnitude	Very slightly increases	Increases more than three orders of magnitude	Even if the intensities of electrical and opti-cal fluctuations are almost constant, r may change noticeably during the aging as the correlated, and uncor-related parts in the total electrical fluctua-tions vary $(S_{\mathrm{el}\;\mathrm{total}}\left(f\right)=S_{\mathrm{el}\;\mathrm{cor}}\left(f\right)+S_{\mathrm{el}\;\mathrm{uncor}}\left(f\right)$)
Optical noise	Decreases by two orders of magnitude			Increases more than one order of magnitude	Slightly increases, the noise level fluctuates up to one order of magnitude	Increases more than one order of magnitude	
Cross-correlation coefficient between electrical and optical fluctuations *r* ^1^	Decreases about 20%	Increases about 30%, very high at (2010–2500)-h period	Increases but follows unstable noise behavior	Minimal changes, follows the optical noise variation		Increases more than 40%	
Phosphor layer	None	The ratio between intensities of yellow and blue parts of optical spectrum increases		None (the ratio between intensities of yellow and blue parts of optical spectrum is almost constant)			

^1^ Here results of the cross-correlation coefficient in frequency range of (10 Hz–20 kHz) are compared.

## Data Availability

The data presented in this study are available on request from the corresponding author.
